# Extradural Primary Malignant Spinal Tumors in a Population Younger than 25 Years: An Ambispective International Multicenter Study on Onco-Surgical Outcomes

**DOI:** 10.3390/cancers15030845

**Published:** 2023-01-30

**Authors:** Alexander C. Disch, Stefano Boriani, Alessandro Luzzati, Laurence D. Rhines, Charles G. Fisher, Aron Lazary, Ziya L. Gokaslan, Dean Chou, Michelle J. Clarke, Michael G. Fehlings, Klaus-Dieter Schaser, Nicole M. Germscheid, Jeremy J. Reynolds, The AO Spine Knowledge Forum Tumor

**Affiliations:** 1 University Center for Orthopedics, Trauma & Plastic Surgery, University Comprehensive Spine Center (UCSC), University Hospital Carl Gustav Carus Dresden at the TU Dresden, 01307 Dresden, Germany; 2 I.R.C.C.S. Istituto Ortopedico Galeazzi, 20161 Milan, Italy; 3 Department of Neurosurgery, MD Anderson Cancer Center, Houston, TX 77030, USA; 4 Department of Orthopaedics, Faculty of Medicine, The University of British Columbia, Vancouver, BC V5Z 1M9, Canada; 5 National Center for Spinal Disorders, 1126 Budapest, Hungary; 6 Department of Neurosurgery, The Warren Alpert Medical School of Brown University, Rhode Island Hospital and The Miriam Hospital, Providence, RI 02903, USA; 7 Department of Neurosurgery, The UCSF Spine Center, University of California, San Francisco, CA 94143, USA; 8 Department of Neurosurgery, Mayo Clinic, Rochester, MN 55902, USA; 9 Department of Surgery Halbert Chair, Spinal Program University of Toronto, Toronto Western Hospital University Health Network, Toronto, ON M5T 2S8, Canada; 10 AO Spine Knowledge Forum Tumor, AO Spine, 7270 Davos, Switzerland; 11 Oxford Spinal Surgery Unit, Oxford University Hospitals, Oxford OX3 7LE, UK

**Keywords:** extradural malignant primary tumor, spinal tumor, adolescent, children, aggressive resection, surgical outcome

## Abstract

**Simple Summary:**

Extradural primary malignant spinal tumors in the younger population are a rarity. Their diagnosis can cause an ordeal for the patients as well as their families. Specialist multidisciplinary sarcoma management is mandatory. Only limited outcome information on interdisciplinary treatment is available. The aim of this study is to report on the clinical outcomes of young patients who received surgery for an extradural primary malignant spinal tumor. This is the first and largest multicenter cohort of surgically treated extradural malignant spinal tumors in young patients. The results underline the value of surgery in a multidisciplinary concept with the intent to cure using EA resections to achieve better overall survival and low local recurrence rates. Due to the necessary experience for pediatric oncology care and specialist spinal oncology surgery, primary malignant spinal tumor treatment in young patients must be centralized.

**Abstract:**

Extradural malignant primary spinal tumors are rare and outcome data, especially for younger patients, is limited. In a worldwide (11 centers) study (Predictors of Mortality and Morbidity in the Surgical Management of Primary Tumors of the Spine study; ClinicalTrials.gov Identifier NCT01643174) by the AO Spine Knowledge Forum Tumor, patients surgically treated for primary tumors of the spine between 1992 and 2012, were retrospectively analyzed from a prospective database of their medical history. Medical history, tumor characteristics, diagnostics, treatments, cross-sectional survival, and local recurrences were analyzed. Sixty-eight cases (32 f; 36 m), at an average age of 18.6 ± 4.7 years at the time of diagnosis, were identified (median follow-up 2.9 years). The most common entities were Ewing’s sarcoma (42.6%). Of the patients, 28% had undergone previous spine tumor surgery in another center (84% with intralesional margins). Resection was considered “Enneking appropriate” (EA) in 47.8% of the cases. Of the patients, 77.9% underwent chemotherapy and 50% radiotherapy. A local recurrence occurred in 36.4%. Over a third of patients died within a 10-year follow-up period. Kaplan-Meier-analysis demonstrated statistically significant overall survival (*p* = 0.007) and local recurrence rates (*p* = 0.042) for tumors treated with EA surgery versus Enneking inappropriate surgery. Aggressive resection of extradural primary malignant spinal tumors combined with adjuvant therapy reveals low local recurrence rates and better outcomes overall in younger patients.

## 1. Introduction

Primary malignant tumors, especially spinal manifestations, are rare in comparison to the overall number of primary tumors affecting the musculoskeletal system [1,2,3,4]. In contrast to secondary spinal lesions, incidences have been stable over recent decades [5]. Treatment is challenging due to complex surgical anatomy and often a poor response to chemotherapy and radiation.

For non-metastasized tumors (Enneking IA-IIB) [6,7], tumor resection, with or without standardized neo-/adjuvant protocols, aims to completely remove all tumor tissue in an enbloc fashion with clear margins [8]. To provide a consistent assessment of the resection success [9], instead of referring to the underlying surgical technique, the achieved surgical margins are (in accordance with the Enneking classification) expressed by the terms Enneking-appropriate (EA) or Enneking-inappropriate (EI) [10]. EA resections in combination with adjuvant therapies have shown better local tumor control (LR) rates, overall survival (OS), and health-related quality of life (HRQOL) in malignant primary spinal tumors [11,12,13,14,15,16]. Therefore, achieving these margins is a major goal and must be weighed against the potential surgical morbidity and mortality, a complex, and stressful shared decision-making process.

For young patients and their families, the diagnosis of a primary malignant spinal tumor is an ordeal during which they face a long and extensive course of therapeutical steps in a multidisciplinary treatment setting. Compared to adult patients, oncological management for primary malignant spinal tumors in younger patients profoundly differs, while two age groups are differentiated in the literature: (1) from birth until 14 years of age; and (2) from 15 to 25 years of age [17,18]. However, similar treatment protocols are used in children and young adults up to their mid-twenties [19].

Especially in young patients, the concept of aggressive enbloc resections with neurological and extensive soft tissue sacrifice remains controversial due to a paucity of literature [20]. Whether less aggressive surgical approaches are justified, especially in young patients, is a constant matter of debate [21,22].

The aim of the current study was to report on the clinical outcomes in patients under the age of 25 years, that were enrolled in the Predictors of Mortality and Morbidity in the Surgical Management of Primary Tumors of the Spine study (ClinicalTrials.gov Identifier NCT01643174), a multi-center ambispective analysis of onco-surgical results [10]. The Knowledge Forum Tumor of the AOSpine is mainly engaged in prospective data acquisition and analysis, e.g., from the PTRON study framework. A further scientific goal was to identify and specify future study questions for the analyses of results from prospective cohorts.

## 2. Materials and Methods

### 2.1. Design and Patients

**Figure 1:**
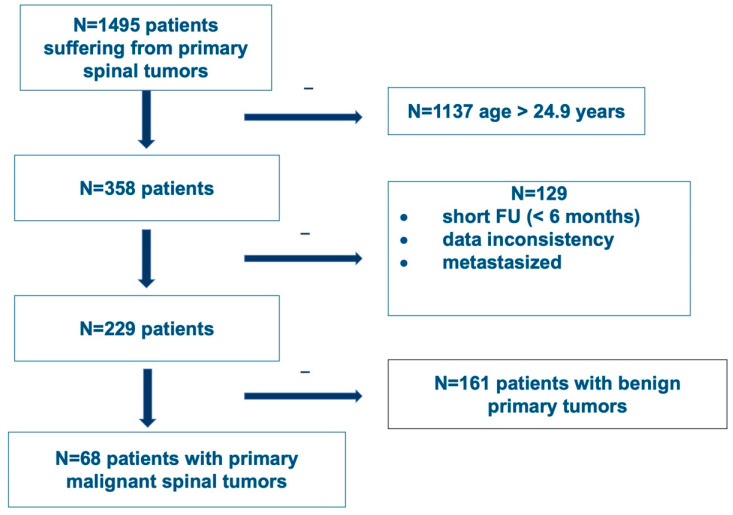
Flowchart of patient inclusion from the AO Spine Knowledge Forum Tumor’s Predictors of Mortality and Morbidity in the Surgical Management of Primary Tumors of the Spine study (CinicalTrials.gov Identifier NCT01643174).

In an ambispective, multicentric review of prospectively collected data, initiated by the AO Spine Knowledge Forum Tumor, all patients admitted to one of the participating centers with a diagnosis of a primary spinal tumor later treated surgically with at least one follow-up detectable were included in the Predictors of Mortality and Morbidity in the Surgical Management of Primary Tumors of the Spine study database. Patients having a metastatic tumor of the spine or having a primary spinal cord tumor were excluded. For further analysis, patients less than or equal to 24.9 years of age at the time of diagnosis with a follow-up greater than 6 months (and no data inconsistencies or tumors that metastasized) were included. Ten international spine centers in Europe and North America included patients treated from 1992 until 2012. Out of the 1495 patients collected, 68 patients under the age of 25 years were diagnosed with a malignant primary spinal tumor and included (Figure 1). Tumors were classified according to the 4th edition of the WHO classification. Data were centralized and captured using a web-based software platform (REDCap, Vanderbilt University, Nashville, TN, USA). Ethics approval was obtained at each of the participating centers.

Patient data included demographics, patient clinical and tumor characteristics, treatment details of surgery, applied chemotherapy and radiation therapy as well as oncological outcome parameter. The cohort was further sub-analyzed according to the achieved resection success and grouped into EA and EI surgical resections and time points of chemo and radiation therapies.

### 2.2. Neurological Status

Preoperative Frankel and/or American Spinal Injury Association Impairment Scale (ASIA) scores were recorded. In cases where both scores were recorded, and a discrepancy occurred, the most severe score was chosen.

### 2.3. Treatment

All patients included in this analysis underwent spinal tumor resection. The use of embolization to minimize intraoperative bleeding prior to these surgeries was recorded. A broad range of surgical approaches and techniques was applied to reach the planned resection goals. The mode and sequence of the approaches used, the use of a fixation, and the need to sacrifice neurological structures (cord/cauda/roots) in terms of radicalness, were analyzed. Applied adjuvant therapies and their timing were added to the database.

According to the Enneking classification and recommendations [6,7,9], an onco-surgical sufficient resection margin is defined as EA, and finally confirmed by the histopathologist. If the final pathology margin was not matching the Enneking recommended surgical margin, it was defined as EI. Therefore, wide and marginal margins in the pathologists’ final statement was considered EA, while intralesional margins and all patients with previous surgeries, independent from the reached margin, were considered EI [10].

### 2.4. Statistics

Patient data analysis was performed using descriptive statistics. Student’s *t*-test and Mann-Whitney U test were used, as appropriate, for continuous variables. *Χ*^2^-tests (Pearson and Fisher exact tests) were used for categorical variables. Survival and LR were illustrated by Kaplan-Meier curves. Significance was defined at a *p*-value of 0.05. The STATA software was used for statistical analyses (version 12.0, College Station, TX, USA).

## 3. Results

### 3.1. Patients

There was a total of 68 patients (32 female; 36 male) diagnosed with an extradural primary malignant tumor with a mean age at the time of diagnosis of 18.6 (±4.7) years and 19.3 (±4.8) years at the time of surgery (Table 1).

Most patients presented with pain at diagnosis (95.3%). Pathological fractures were found in 12.9% of the cases. Preoperative neurological status according to the Frankel and/or ASIA score was A/B/C/D/E in 1.5/3.1/9.2/20.0/66.2% of patients, respectively.

The final diagnosis was achieved by open, CT-trocar, or intraoperative biopsy in 85.3% of cases. 19 (27.9%) patients were previously treated surgically and were—in addition to cases that underwent biopsy elsewhere—categorized as EI.

Tumor diagnosis, according to the histopathological results, is displayed in Table 2, where 29 (42.6%) cases were diagnosed as Ewing’s Sarcoma. Tumor grade according to the Enneking classification was Ib/IIa/IIb in 7.4/10.3/82.4 % of cases, respectively.

Tumors were located at the mobile spine in 47 (69.1%) cases. The distribution regarding the spinal region was cervical/ thoracic/ lumbar/ sacral region in 13.5/25.0/38.5/23.1% of cases, respectively. Tumor volume according to spinal tumor imaging was equal to, or exceeded, 5 cm^3^ in 54 (94.7%) cases. Multilevel (≥2 vertebrae) involvement was seen in 45 (66.2%) cases (Table 2).

### 3.2. Treatment

To reach adequate oncological resection, a sacrifice of spinal cord (*N* = 1) or cauda equina (*N* = 2) was necessary. Nerve root resection was performed in 37(56.9%) cases. Tumor resection without spinal column reconstruction was performed in 26.5% of cases.

Based on the pathological results of the intraoperative specimen, wide or marginal resections were achieved in 59.7% (*N* = 40), and intralesional in 40.3% (*N* = 27) of cases. Including patients with previous surgeries—cases with biopsies and intralesional surgeries elsewhere—52.2% (*N* = 35) of patients were consequently categorized as EI (Table 3).

According to the oncological protocols, most of the patients (77.9%) underwent adjuvant therapy, where 23.5% were preoperative, 11.8% were postoperative, and 35.3% were at both time points. The timings of chemotherapy pre- or postoperatively did not show an effect on LR or OS rates (*p* > 0.05; Table 4 and Table 5).

Preoperative radiation therapy was performed in 25% of cases, 23.5% following surgery, and 1.5% at both time points. Most of the patients (85.3%, *N* = 29) received conventional radiation therapy (EBRT). The timing of radiation therapy in relation to the surgical intervention also showed no influence on LR and OS (*p* > 0.05).

**Figure 2:**
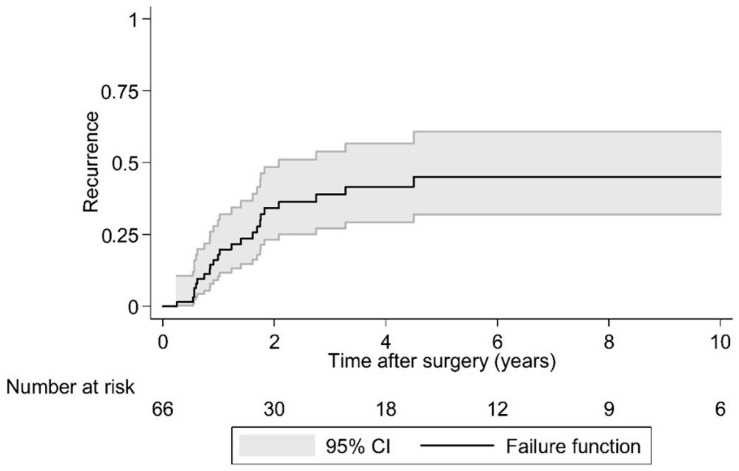
Kaplan-Meier analysis of time to first local recurrence following surgery. Note: There are 66 cases in this analysis because in two cases the timing of local recurrence was unknown.

**Figure 3:**
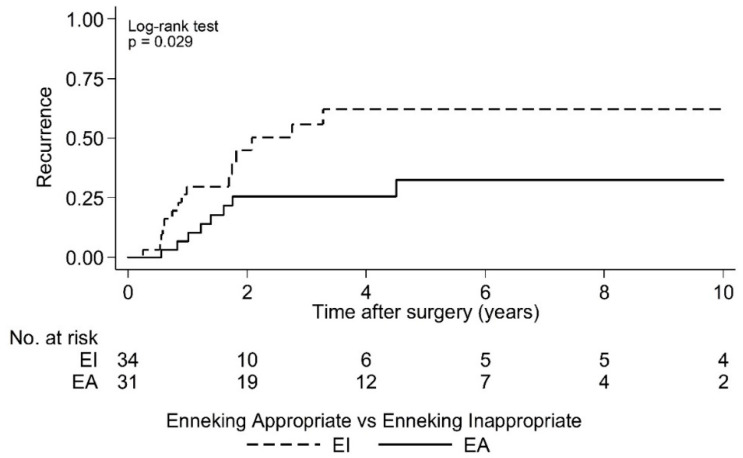
Kaplan-Meier analysis of time to first local recurrence by Enneking appropriateness. EI: Median time to first local recurrence = 2.1 years postoperative [95% CI 1.7, -]; EA: Median time to first local recurrence = not reached. Note: There are 65 cases in this analysis because the timing of local recurrence was unknown for two cases.

**Figure 4:**
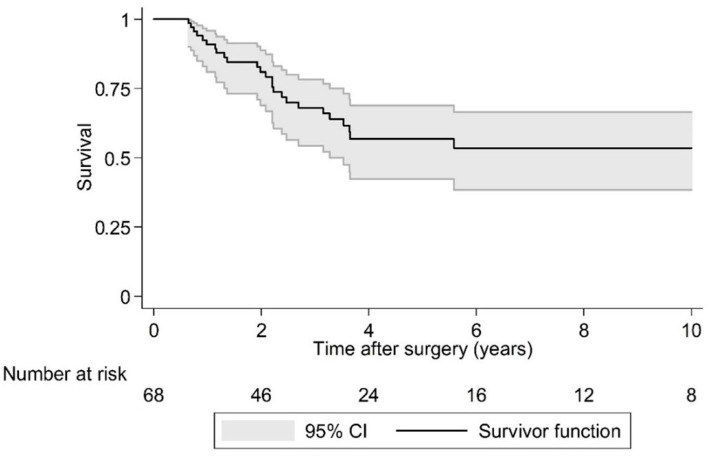
Kaplan-Meier analysis of overall survival.

**Figure 5:**
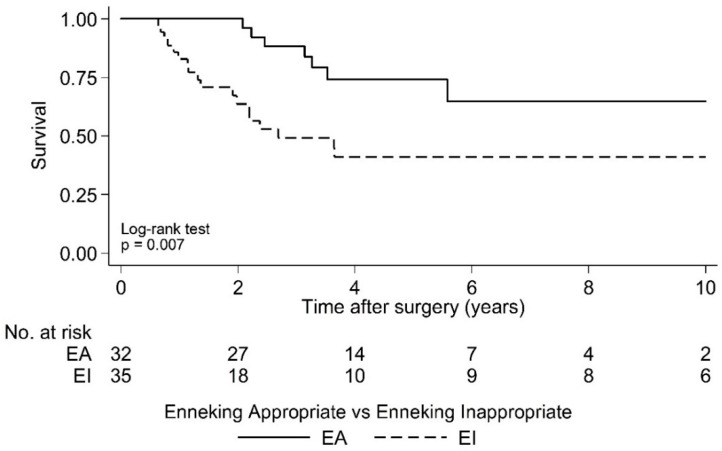
Kaplan-Meier analysis of overall survival following surgery by Enneking appropriateness. EI: Median Survival = 2.6 years postoperative [95% CI 1.9, -]; EA: Median Survival = not reached.

Postoperative LR was seen in 36.4% (*N* = 24) of cases over a 10 year period. As the largest group of aggressive tumor entities, Ewing sarcomas demonstrated a local relapse rate of 37.0%. The median time to the first LR in the EI group was 2.1 years (95% CI 1.7, -) postoperatively and that was significantly earlier compared to the EA group (log rank test *p* = 0.029; Figure 2 and Figure 3). Overall survival showed 63.6% (*N* = 29) of cases with no evidence of disease (NOD)/alive with disease (AWD), and 36.4% dead of disease (DOD) over a 10-year period. Out of the 29 patients suffering from a Ewing sarcoma, the median survival was 3.3 years. Survival analysis by surgically achieved Enneking appropriateness showed a median survival of the EI group of 2.6 years (95% CI 1.9, -) postoperatively and differed therefore significantly from the EA group results (log rank test *p* = 0.007; Figure 4 and Figure 5). Table 6 outlines the key parameters for each histotype regarding the overall number/percentage, tumor grade according to the Enneking classification, resection result, LR and OS.

## 4. Discussion

Modern oncological treatment concepts for extradural primary malignant spinal tumors aims to achieve standardization, but individual case constellations do influence the course of events, especially surgical management [21,23]. The age of patients does play a major role, among others, regarding the inclusion into corresponding treatment protocols, decision-making for surgical interventions, and possible emotional bias influencing the treatment team when children or young adults are affected [1,24,25].

The presented ambispective study of the AO Spine Knowledge Forum Tumor represents the results of one of the largest multicenter cohorts of patients under the age of 25 years surgically treated for extradural primary malignant spinal tumors. Enneking-appropriateness, as a resection result of an aggressive surgical strategy in combination with adjuvant protocols, was demonstrated to also be a primary outcome determent factor, significantly correlating to improved local control and overall outcome in a younger patient group.

Spinal sarcoma entities that are indicated for surgical treatment are well described in common literature [6,8,26] and the results of this study are in accordance with them. In the presented young patient group Ewing´s sarcoma, osteo and chondrosarcomas were the most common tumors in a heterogeneous distribution. Manifestation characteristics during childhood and adolescence are evenly and numerously published [1,5,17,19,22,27], and clinical appearance is typically linked to the underlying biological activity [6,9,21]. Bone sarcomas derive from various cell lines of different origins, mesenchymal tissues, or non-epitheloidal mesodermal, and ectodermal or neuroectodermal germ layers. Oncological treatment for systemic and local control profoundly differ and is consequently processed by sarcoma-specific standardized, internationally consented protocols, with surgical interventions embedded in the treatment plan if recommended [8]. However, even with variations in tissue origin, the general biological behavior of primary malignant tumors in relation to the surrounding tissue layers can be classified, and as a result, tumor aggressiveness is successfully graded in relation to compartmental borders. In accordance with other publications of this group, surgical outcome results of spinal primary tumor treatment, aggressive resections are, by considering biological activity (displayed by the Enneking classification and resection recommendation), inevitably related to a successful onco-surgical treatment [11,13,14,16]. LR in bone sarcoma patients is known to be a predictor for less favorable outcomes. In this study, patients with EI resections evidenced a continuous increase in LR in the first 3–5 years following surgery, while LR in EA patients did occur in the first 2 years after surgery. More than half of the EI patients died during the investigation period with the remaining patients stable after 4 years. In EA patients, a parallel survival curve progression was found, but on a significantly higher level.

Age-dependency was shown to be a relevant factor for sarcoma treatment outcomes, even in cohorts of children and adolescents [21,24,28]. The results of the current study underline the importance of a comprehensive EA management plan from the outset (including biopsy and staging) where the diagnosis of a primary extradural tumor is a possibility, especially in a young patient group less than 25 years of age. Misinterpretation of symptoms, and underestimation of unspecific findings, especially pain [29], paired with very low incidences, significantly decreases the rate of early tumor detections. As a result, delayed sarcoma diagnosis is a commonly reported risk [26,30]. Not only in younger patients, the suspicion of a tumor makes a consequent diagnostic workup, classification, and treatment strategy mandatory. Algorithmic case workup provides a maximized chance for subsequent surgical successes, allowing significantly better LC rates including an acceptable HRQOL in a majority of cases [31].

However, in contrast to that mentioned doctrine, the results from the global cohort unexpectedly demonstrated a high number of patients with Enneking-inappropriate (EI) surgeries. Even treated in highly specialized international spine tumor centers, the onco-surgical outcome did not match the recommendations in more than half of the investigated cases. Thereby, patients with the result of an EI-resection should be subdivided into two larger groups. First, some patients in the cohort received an initial intervention outside the institution which was later responsible for the definitive spinal surgical treatment and consequently graded as EI. That included, on the one hand, diagnostic procedures like inadequately performed biopsies. The high heterogeneity of previous biopsies was found in the mentioned study in terms of technical aspects and approaches. Especially, interdisciplinary pre-interventional planning still seems to be a matter of concern to avoid unforced transgression of unaffected compartments and tumor cell seeding along the biopsy tract [32,33,34].

Furthermore, half of the EI patients underwent previous surgeries elsewhere for treatment of the spinal tumor. Non-virgin presentation and previous “out-of-center” management still do exist in relevant numbers. In general, these cases have been shown to result in more complicated courses, arising from inadequate diagnostic workup, and delayed diagnoses, to higher surgical complication rates and less favorable onco-surgical outcomes [29,35,36]. Even if successful revision surgeries are described for some entities [37,38], secondary treatment in an experienced spinal oncology center is associated with a more complicated course. The first diagnostic and treatment approach has been shown to set the patient’s fate in terms of LR rates and overall outcome [8,39].

However, even when treated in one of the primary study centers, some patients showed a less favorable surgical EI resection. Different reasons might have influenced this result. Presentation with neurological deficits put multidisciplinary teams time wise under pressure and can usually disturb the regular workup process (33.8% of patients in that series with preoperative Frankel and ASIA Score D or worse). In cases with mild neurological deficits and high-grade tumors, the prompt start of neoadjuvant therapies under corticosteroids can avoid emergency interventions and have shown to significantly improve neurological deficits [22]. Later, definitive surgery can be carried out regularly during the surgical window [40]. However, a complex situation arises when young patients initially present with significant neurological deterioration. As emergency decompressions of nerval structures are, compared to aggressive surgery, not usually orientated to compartmental borders, uncontrolled tumor transgression will lead to local tumor cell spread and seeding. While neurological deficits might improve, the onco-surgical constellation worsens. Whenever possible, and even under difficult emergency circumstances, objective multidisciplinary decision-making must be established [8,25,41]. Malignant primary spinal tumor diagnosis is an ordeal for young patients and their relatives, giving distinction on several years or even decades of the following lifespan [25]. Open, repetitive information transfer and sustained interaction with patients and their families are prerequisite to allowing the best possible long-term outcome [22]. In addition, a successful onco-surgical outcome itself is clearly related to improved health-related quality of life [31,36,42].

For some of the EI cases, it can only be speculated retrospectively what individual reasons have promoted inappropriate resections. Unexpected intraoperative surgical technical issues, intraoperative complications, or irresectability might have limited successful tumor removal [35]. Concerns prior to aggressive surgery (e.g., sacrificing nerval roots) and associated disability in young patients are common, and weighing out function versus complete tumor removal is difficult to accept for patients, relatives, and physicians [22].

Various limitations exist for this analysis and careful interpretation of this data is recommended. With primary malignant spinal tumors, the origin, histopathological and radiological appearance, and biological behavior differ severely, and all are included in a highly heterogeneous group. All mentioned entities are thereby rarities, making standardized workup even more difficult. Advanced statistical analysis and modeling is not possible with low patient numbers. Due to the ambispective nature of the study and the short follow-up periods, especially in a multicentric approach, data availability is limited (e.g., missing postoperative AIS). The evaluation of the outcome data should be interpreted with caution due to limited follow-up data. Whilst there are variances in the appearance and behavior between tumor pathologies, there are obviously many similarities between the cohorts chosen to be included to the current study. These similarities have driven the original Enneking Classification and the subsequent recommendations regarding surgery. Despite the described shortcomings, this paper displays, for the first time, results from the largest cohort of young patients surgically treated to date and thereby contributes significantly to literature across the breadth of these spinal tumors. The results provide a solid basis for planning and further analyzing in future prospective studies by this group.

## 5. Conclusions

To our knowledge, this is the largest cohort, and first time such data has been presented for patients younger than 25 years of age. The results revealed that patients younger than 25 years of age suffering from extradural primary malignant spinal tumors undergoing successful aggressive resections show significantly better oncological outcomes in terms of LR rates and OS. The outcome was primarily related to the biological activity expressed by the Enneking classification. The majority of tumors surgically treated in this study were graded as type IIb according to the Enneking classification. In contrast to some benign tumor constellations [43,44,45], resection recommendations, including adequate adjuvant therapy and later treatment success, were clearly related and independent from the underlying entity.

## Figures and Tables

**Table 1:** Summary of patient demographic and clinical characteristics.

Variable	N (%) or Mean ± Standard Deviation
Gender (*n* = 68)	
Female	32 (47.1)
Male	36 (52.9)
Age at time of diagnosis (years) (*n* = 68)	18.6 ± 4.7
Age at time of surgery (years) (*n* = 68)	19.3 ± 4.8
Pain at Diagnosis (*n* = 64)	
No	3 (4.7)
Yes	61 (95.3)
Pathologic Fracture at Diagnosis (*n* = 62)	
No	54 (87.1)
Yes	8 (12.9)
Previous Spine Tumor Operation (*n* = 68)	
No	49 (72.1)
Yes	19 (27.9)
Intralesional	16 (84.2)
Unknown	3 (15.8)
Preoperative Frankel and ASIA Score * (*n* = 65)	
A	1 (1.5)
B	2 (3.1)
C	6 (9.2)
D	13 (20.0)
E	43 (66.2)

* When a discrepancy between ASIA and Frankel scores occurred, the more severe score was chosen.

**Table 2:** Summary of malignant spinal tumor characteristics.

Variable	N (%) or Median (p25, p75)
Diagnosis (*n* = 68)	
Chordoma	5 (7.4)
Chondrosarcoma	12 (17.6)
Osteosarcoma	18 (26.5)
Ewing’s sarcoma	29 (42.6)
MPNST	4 (5.9)
Tumor Volume Ellipsoid Body (cm^3^) * (*n* = 57)	62.8 (20.9, 169.6)
<5	3 (5.3)
≥5	54 (94.7)
Spinal level (*n* = 68)	
Mobile	47 (69.1)
Fixed	21 (30.9)
Level by Cervical, Thoracic, Lumbar, Sacral (*n* = 52)	
Cervical	7 (13.5)
Thoracic	13 (25.0)
Lumbar	20 (38.5)
Sacral	12 (23.1)
No. of Vertebral Levels Spanned by the Tumor (*n* = 68)	
1	23 (33.8)
≥2	45 (66.2)
Tumor Grade [Enneking Classification] (*n* = 68)	
Ib	5 (7.4)
IIa	7 (10.3)
IIb	56 (82.4)

* Tumor Volume Ellipsoid Body = π/6 × height × width × depth.

**Table 3:** Summary of treatment details.

Preoperative Embolization (*n* = 32)	
No	55 (84.6)
Yes	10 (15.4)
Fixation Used (*n* = 68)	
Anterior	1 (1.5)
Posterior	41 (60.3)
Both	8 (11.8)
None	18 (26.5)
Neurology Sacrificed: Cord (*n* = 66)	
No	65 (98.5)
Yes	1 (1.5)
Neurology Sacrificed: Cauda Equina (*n* = 66)	
No	64 (97.0)
Yes	2 (3.0)
Neurology Sacrificed: Nerve Roots (*n* = 65)	
No	28 (43.1)
Yes	37 (56.9)
Pathology result from the surgical specimen (*n* = 67)	
Wide or marginal	40 (59.7)
Intralesional	27 (40.3)
Enneking appropriateness (*n* = 67)	
EA	32 (47.8)
EI	35 (52.2)
Adjuvant therapy (*n* = 68)	
No	15 (22.1)
Yes	53 (77.9)
Timing of chemotherapy (*n* = 68)	
Preop	16 (23.5)
Postop	8 (11.8)
Both	24 (35.3)
Neither (no chemo)	20 (29.4)
Timing of radiation therapy (*n* = 68)	
Preop	17 (25.0)
Postop	16 (23.5)
Both	1 (1.5)
Neither (no radiation)	34 (50.0)
Type of Radiation Therapy given (*n* = 34)	
Conventional	29 (85.3)
IMRT	2 (5.9)
Radiosurgery	2 (5.9)
Proton Beam	1 (2.9)
Local recurrence over 10 years postoperative (*n* = 66)	
No	42 (63.6)
Yes	24 (36.4)
Survival over 10 years postoperative (*n* = 66)	
Alive	42 (63.6)
Dead	24 (36.4)

**Table 4:** Local recurrence and overall survival rates in relation to the timing of chemotherapy.

**Timing of Chemotherapy**	**Local Recurrence**	
	**No N (%)**	**Yes N (%)**
**Preop**	10 (62.5)	6 (37.5)
**Postop**	3 (37.5)	5 (62.5)
Fisher’s exact test = 0.390		
**Timing of Chemotherapy**	**Death**	
	**No N (%)**	**Yes N (%)**
**Preop**	9 (56.3)	7 (43.8)
**Postop**	4 (50.0)	4 (50.0)
Fisher’s exact test = 1.000		

**Table 5:** Local recurrence and overall survival rate in relation to the timing of radiation therapy.

**Timing of Radiation Therapy**	**Local Recurrence**	
	**No N (%)**	**Yes N (%)**
**Preop**	12 (70.6)	5 (29.4)
**Postop**	10 (66.7)	5 (33.3)
Fisher’s exact test = 1.000		
**Timing of Radiation Therapy**	**Death**	
	**No N (%)**	**Yes N (%)**
**Preop**	9 (56.3)	7 (43.8)
**Postop**	4 (50.0)	11 (45.8)
Fisher’s exact test = 1.000		

**Table 6:** Key parameters for each histotype regarding the overall number/percentage, tumor grade according to the Enneking classification, resection result, LR and OS.

Diagnosis	N (%)	Tumor Grade [Enneking Classification] (*n* = 68) *N* (%)	Enneking Appropriateness (*n* = 67) *N* (%)	Local Recurrence Over 10 Years Postoperative (*n* = 66) *N* (%)	Survival Over 10 Years Postoperative (*n* = 66) *N* (%)
Chordoma	5 (7.4)	Ib = 2 (40)	EA = 4 (80)	No = 2 (40)	Alive = 3 (60)
		IIb = 3 (60)	EI = 1 (20)	Yes = 3 (60)	Dead = 2 (40)
Chondrosarcoma	12 (17.6)	Ib = 3 (25)	EA = 8 (66.7)	No = 9 (75)	Alive = 10 (83.3)
		IIb = 9 (75)	EI = 4 (33.3)	Yes = 3 (25)	Dead = 2 (16.7)
Osteosarcoma	18 (26.5)	IIb = 18 (100)	EA = 8 (44.4)	No = 12 (66.7)	Alive = 10 (55.6)
			EI = 10 (55.6)	Yes = 6 (33.3)	Dead = 8 (44.4)
Ewing’s sarcoma	29 (42.6)	IIa = 5 (17.2)	EA = 11 (39.3)	No = 17 (63)	Alive = 15 (55.6)
		IIb = 24 (82.8)	EI = 17 (60.7)	Yes = 10 (37)	Dead = 12 (44.4)
MPNST	4 (5.9)	IIa = 2 (50)	EA = 1 (25)	No = 2 (50)	Alive = 4 (100)
		IIb = 2 (50)	EI = 3 (75)	Yes = 2 (50)	Dead = 0 (0)

## Data Availability

Data Management and availability.
